# Does bribery increase maternal mortality? Evidence from 135 Sub-Saharan African regions

**DOI:** 10.1371/journal.pgph.0000847

**Published:** 2023-12-04

**Authors:** Veronica Toffolutti, Eugenio Paglino, Alexandros Kentikelenis, Letizia Mencarini, Arnstein Aassve

**Affiliations:** 1 Health Economics and Policy Research Unit, Wolfson Institute of Population Health, School of Medicine and Dentistry, Queen Mary University of London, London, United Kingdom; 2 “Carlo F. Dondena” Centre for Research on Social Dynamics and Public Policies, Bocconi University, Milan, Italy; 3 Population Studies Center, University of Pennsylvania, Philadelphia, Pennsylvania (PA), United States of America; 4 Department of Social and Political Science, Bocconi University, Milan, Italy; BRAC University, BANGLADESH

## Abstract

About 295,000 women died globally during and following pregnancy and childbirth in 2017. Two-thirds of these deaths occurred in Sub-Saharan Africa. By linking individual and regional data from 135 regions in 17 Sub-Saharan African countries over the period 2002–2018 this study explores how bribery affects maternal mortality in Sub-Saharan Africa. Our results show that the percentage of people who had first-hand experience in bribery is significantly and positively associated with pregnancy related deaths. We find that a 10 p.p. increase in the prevalence of bribery is associated with up to 41 [95% CI: 10–73] additional deaths for every 1,000 pregnancy-related deaths. However, the healthcare system quality appears to be an important moderator. To reduce maternal mortality, policy makers should not only increase investments in healthcare, they need also to implement measures to combat corruption.

## 1. Introduction

Reducing maternal mortality (MM) has been at the forefront of the global health agenda for decades. The Millennium Development Goals elevated it to a top priority. Despite progress the associated target for 2015 was only met by five countries [1]. The Sustainable Development Goals (SDGs) codified the international community’s commitment to further reducing the maternal mortality ratio (number of maternal deaths per 100,000 live births) to less than 70 by 2030. Again, progress towards this target has been lagging: about 295,000 women died during and following pregnancy and childbirth in 2017; roughly two-thirds of these deaths occurring in Sub-Saharan Africa (SSA) [2]. Approaches for how to best reduce MM have focused overwhelmingly on the role of clinical interventions. For example, concerted efforts were made to increase the number of skilled attendants and to increase, too, quality obstetric care [3, 4]. These kinds of interventions are important for addressing the proximal, biomedical aspects of maternal health, including questions around the availability and the quality of health services. But the slow progress in improving MM is symptomatic of the broader problem of health care access in lower income countries. Round six of the Afrobarometer survey shows that nearly half of all respondents reported foregoing necessary healthcare, and 4 out of 10 of those who received care in the previous year found it difficult to get treatment [5, 6]. The reasons are many: stigmatization [7]; lack of education [8]; transportation difficulties [8–11]; and direct financial barriers, such as user fees and out-of-pocket or informal payments [12]. As explained by Lewis (2000), ‘informal payments…provide a means by which corrupt public servants can ensure or maximize their income, evade taxes, and effectively “beat the system” and consequently are a form of systemic corruption’ [13]. There is a growing debate about the role of informal payments and whether it represents a form of corruption (see Schaaf and Topp (2019) review on the topic [14]). As suggested by Schaaf and Topp (2019) informal payments are “absolutely necessary to keep the facility operating or to deliver a service” [14]. It does not, however, necessarily imply corruption. This study follows up on this line research with a focus on questions asked in the Afrobarometer survey that explicitly make reference to “bribery” and that are currently used by the Global Corruption Barometer to assess how corruption affects individuals’ views and experience in Africa [15, 16].

The healthcare sector is particularly exposed to corruption. Significant public funds, and the wide array of actors involved—from policymakers, to healthcare providers, to suppliers—all make health systems vulnerable to corrupt practices [17]. Not surprisingly, corruption associates with poorer health, including lower levels of life expectancy [18]; higher mortality rates [18–20] lower levels of subjective health [18]; low immunization rates [20]; and poor management of chronic conditions [21]. Corruption is, however, a broad concept and not straight forward to define, let alone constructing reliable measurements [20]. One of the World Bank’s six WGI dimensions of governance includes *corruption control*, though without actually providing a definition of corruption itself. The United Nations do not provide a definition of corruption either. Instead, they list a set of practices deemed as being corrupt [22]. Drawing on these practices, Gaitonde *et al*. (2016) defined corruption as “the abuse or complicity in abuse, of public or private position, power or authority to benefit oneself, a group, an organization or others close to oneself; where the benefits may be financial, material or non-material” [22]. What is clear, however, is that corruption is a global problem. Transparency International’s 2013 Corruption Barometer survey reported that over half the respondents in 42 of the 109 countries viewed their health systems as being corrupt or very corrupt [23]. In light of this, nearly 25% of respondents from 119 countries reported that they had paid a bribe to access public services in the previous 12 months, between March 2014 -January 2017 [5].

Despite the relevance of corruption for the health sector, we still know little about how it affects MM. One main reason is the lack of survey data that, simultaneously, measure both MM and corruption. For instance, the individual survey most used to measure MM, is the Development and Health Surveys (DHS). However, the DHS does not provide measures of corruption. In contrast, surveys adept to measure corruption, such as the Afrobarometer, cannot be used to measure MM. The Afrobarometer survey offers several measurement options, however. Frequently corruption is proxied by asking respondents about their *perception* of corruption, and this is readily available in the Afrobarometer. Perceptions are of course subjective and potentially a noisy measure. But the Afrobarometer also includes measures of bribery [24, 25]. The measure is more objective as it refers to respondents’ actual experience with bribery. As a result, bribery as a form of corruption has received growing attention from scholars, especially for its role in healthcare systems in developing countries [26–29]. The literature suggests a negative correlation, meaning that health access is lower where bribery is more prevalent [27, 29]. However, in a few cases bribery is associated with a better continuous relationship between patients and healthcare workers [30]. From an economic point of view the association can, therefore, have both a negative and a positive effect on the healthcare supply. In fact, financial incentives can stimulate staff to work more efficiently, which, in turn, will increase the quality of their work [31]. Conversely, some theories suggest that workers might focus on the quantity: more patients in this case, undermining work quality [32]—and in some extreme cases—the quality might also be intentionally reduced to induce more payments [33].

This article fills an important gap by investigating the association with MM and bribery by using data for 470,229 pregnancies from 135 regions in 17 SSA countries between 2002 and 2018. In so doing, this paper provides three major contributions to the academic and policy debates on global health. First, we offer new evidence on how structural determinants, in this case corruption measured by bribery, affect MM [34–36]. Second, we provide evidence on the time-trend of MM at regional level for 17 SSA for a period of over 15 years, therefore connecting the unique nature of the SDGs and their interlocking goals and targets. Third, our study is, to the best of our knowledge, the first one that directly connects within country (and over time) variation in corruption (again through measuring the prevalence of bribery), with the prevalence of MM. This is done by harmonizing the sub-national regions as provided by the DHS (where MM is measured) and the same sub-national regions provided in the Afrobarometer surveys (from where we measure corruption). Whereas this does not capture the prevalence of corruption for particular health care units, it does give a measure of the prevalence of bribery in the sub-national regions where individuals are recorded to reside according to the DHS. This is no small detail, since 1) measures of corruption are generally not available in the DHS, and 2) healthcare provision in developing countries is often highly decentralized [37, 38], with potentially large variation in corruption across those regions where the healthcare services are provided.

The paper proceeds as follows we present the previous literature in section 2, the methodological approach in section 3, the results in section 4 and finally we offer our conclusions.

## 2. Previous literature

### 2.1 The methodological approach

#### 2.1.1 Data sources

We linked data on MM and corruption, measured by bribery, for 135 regions covering 17 SSA countries: Benin, Burkina Faso, Burundi, Cameroon, Kenya, Lesotho, Madagascar, Mali, Mozambique, Nigeria, Senegal, South Africa, Tanzania, Togo, Uganda, Zambia, and Zimbabwe.

Data on MM was drawn from rounds four to seven of the DHS [39]. The DHS is an ongoing cross-sectional survey collecting data from national representative samples of households and from women of reproductive age. The dataset provides detailed information on women’s fertility history: it administers an individual questionnaire to women aged 15–49 using the birth history table that records “all the births the respondent has had in the order in which they occurred starting with her first birth” [40]. In parallel, the survey has the advantage of providing a MM module based on the “sisterhood method” [41]. All sampled women are asked about the survival of their siblings (i.e., individuals birthed by the same mother). Each woman is asked a set of questions on any deceased siblings. In the case of a deceased sister, interviewees are asked: “In what year did [*name*] die? How old was [*name*] when she died? Was [*name*] pregnant when she died? Did [*name*] die during childbirth? Did [*name*] die within two months after the end of a pregnancy or childbirth?” We use, as an outcome, a dummy variable equal to 1,000 if the pregnancy resulted in a pregnancy-related death for the mother and 0 otherwise. Our data show that 8.6 out of 1,000 pregnancies (for a total of 4,037 deaths in our sample) resulted in a pregnancy-related death in the period under examination. However, this aggregate figure masks country-specific variations: the rate varies between 2.1‰ in Togo and 17.5‰ in Lesotho (S1 Fig). One caveat is that, while for women who had no complications during pregnancy or delivery, we are able to observe their complete fertility history up to the interview, we cannot do so for deceased mothers. Therefore, the above number tends to overestimate mortality as deceased women’s previous deliveries are not accounted for in the denominator.

The key exposure variable *bribery* is taken from the Afrobarometer surveys [6]. It is measured by the percentage of citizens’ reports of having engaged in bribery. This is calculated for sub-national regional and year specific levels as the number of citizens’ engaged in bribery over the total number of citizens interviewed in that specific region. The Afrobarometer surveys ask each respondent two relevant questions: “How often, if ever, did you have to pay a bribe, give a gift, or do a favour for a government official in order to get the document you needed? Never/ Once/ Twice/ A few times/ Often” and “How often, if ever, did you have to pay a bribe, give a gift, or do a favour for a police officer in order to get the assistance you needed, or to avoid a problem like passing a checkpoint or avoiding a fine or arrest? Never/ Once/ Twice/ A few times/ Often”. We re-coded responses to these questions into dichotomous variables taking the value of 0 if the response was “Never”, and 1 otherwise. For each of the 135 regions (harmonized across DHS surveys) and each year we compute the proportion of respondents who ever paid a bribe. Only the regions with at least 20 observations *per* wave were kept. Overall, the average number of respondents *per* region-year is 498, with a median of 297. On average 30.7% of the respondents had first-hand experience of bribery, with Kenya ranking at the top of this bribery index with an average of 46.9% and South Africa at the bottom with an average of 9.1% (S2a Fig). S3 Fig presents the distribution of bribery in the sampled sub-national regions over time, and S1 Table presents the number of observations available at country and wave level. Table 1 present descriptive statistics at, respectively, pregnancy and region-year level.

**Table 1:** Descriptive statistics.

	Mean	S.D.	Minimum	Maximum
Individua-Level Variables (N = 246,614)				
No Educational Attainment (Numbers represent percentage of the sample)	0.2936	0.4246	0	1
Primary Education (Numbers represent percentage of the sample)	0.3822	0.4012	0	1
Secondary Education or Higher (Numbers represent percentage of the sample)	0.3242	0.3870	0	1
Died During Pregnancy or within Two Months (Numbers represent percentage of the sample)	0.0092	0.0087	0	0.0645
Woman Lives in Rural Area (Numbers represent percentage of the sample)	0.7953	0.3688	0	1
Pregnancy-Level Variables (N = 470,229)				
Delivery at Facility (Numbers represent percentage of the sample)	0.4554	0.4980	0	1
Received Skilled assistance before or during Delivery (Numbers represent percentage of the sample)	0.3023	0.4592	0	1
Age at Delivery	26.5943	6.6896	10	50
Region-Year Level Variables (N = 943)				
First-Hand Experience with Bribery (Numbers represent percentage of the sample)	0.3073	0.1336	0	0.7954
log(GNI Per Capita) PPP, 2011 US$	7.7360	0.5905	6.4400	9.7600
HDI (range: [0,1])	0.4617	0.0841	0.2310	0.7540
GFR (Births per 100,000 women aged 15–49)	203.7723	45.8890	69.9028	296.5675
Number of Pregnancies	498.6522	637.6543	22	5665

Note: Authors’ elaboration on merged data for 17 SSA countries from the DHS surveys and the Afrobarometer for the period 2002–2018. HDI stands for regional Human Development Index. GFR stands for General Fertility Rate. GNI stands for Gross National Income (per capita). Educational attainment, urban-versus-rural residence, whether the baby was delivered at a facility, and antenatal care or skilled delivery assistance are imputed from the modal value for each sub-national-region-cohort, and are attached to every woman or pregnancy in the sample.

To build an alternative measure of corruption, we used four questions on *perceived corruption* in i) governmental officials; ii) police; iii) office of the presidency; and iv) judges and magistracy. Each of the questions are expressed as “How many of the following people do you think are involved in corruption, or haven’t you heard enough about them to say.” Respondents could answer “None”, “Some of them”, “Most of them”, “All of them”, and “Don’t know”. We coded “Don’t know” responses as missing and the remaining four answers numerically from 1 “None” to 4 “All of them”. We, then, computed the first principal component of the N by 4 matrix with each row representing a respondent and each column one of the four corruption questions. Finally, we aggregated the principal component measure to the sub-national region-year level by computing the average value.

To measure the quality of the maternal healthcare system, we computed the proportion of deliveries by sub-national region and year for which the mother answered affirmatively to the question “Did you see anyone for antenatal care for this pregnancy?” and answered "Health personnel” to the question “Who assisted with the delivery?”. To simplify the analysis, we then constructed a binary variable equal to 1 if either antenatal case was received for more than 50% of the pregnancies in a given region-year or 50% of the deliveries in that region-year were assisted by health personnel.

### 2.2 The methods

We use a linear probability model to assess the association of corruption with MM, as follows:

$$MM_{p,i,r,c,t}=\beta_{0}+\beta_{1}Bribery_{r,t}+\beta_{2}{Bribery}_{r,t}^{2}+\beta_{3}X_{p}+\beta_{4}W_{i}+\beta_{5}R_{r,t}+\sum_{t}\beta_{6,t}DeliveryYear_{t}+\sum_{c}\beta_{7,c}Country_{c}+\varepsilon_{r,c,i,t}$$

MM_p,i,r,c,t_ represents a dummy variable equal to 1 if pregnancy p in year t resulted in a pregnancy-related death for woman i, living in sub-national region r of country c, and 0 otherwise. We multiplied all the coefficients by 1,000 instead to ease the interpretation, as maternal mortality rates are expressed out of 1,000 pregnancies. Bribery represents the percentage of people who reported first-hand engagement in bribery in the same region-year. X_p_ represents a vector of pregnancy-related covariates: the mother’s age; urban or rural residence; attendance of skilled health personnel during delivery/termination; and antenatal care during pregnancy. *W*_*i*_ represents a vector of region-cohort covariates attached to woman *i*, namely educational attainment. *R*_*r*,*t*_ represents a vector of region and year of delivery specific covariates, namely the Human Development Index (HDI) [42] and the Gross Fertility Rate (GFR) [43]. *DeliveryYear*_*t*_ is a dummy for delivery year t to control for temporal correlation, for example those due to outbreaks. *Country*_*c*_ is a dummy for country c. Finally, *ε* represents the error component.

We are interested in the sign and the magnitude of coefficients *β*_1_, *β*_2_ This represents the percentage point variation in the probability of dying during pregnancy or doing so within two months of birth to a one percentage point variation in the percentage of people engaging in bribery as measured per-subnational regions across the time periods.

### 2.3 Data imputation

Unfortunately, the DHS provides only limited information about the deceased mother, e.g. age at delivery and number of living children. However, it does provide abundant information on their sisters. The direct imputation of missing values for the deceased mothers by using sister’s information would create artificial correlations between the imputed variables and the dependent variables. Therefore, education and urban-versus-rural residence is imputed from the modal value for each sub-national-region-cohort and attached to every woman in the sample. The same procedure was used for skilled healthcare personnel attending the delivery and place of delivery. We computed the modal value for each region-year of delivery, which was then attached to the recorded pregnancies. In line with the existing literature we assume that the deceased woman lived in the same region as her sister [41, 44].

### 2.4 Ethics approval and consent to participate

This study is based on proprietary geolocated data: DHS and Afrobarometer data. Access to the data was granted by the MEASURE DHS/ICF International, Rockville, Maryland, USA https://dhsprogram.com/Methodology/GPS-Data.cfm and www.afrobarometer.org.

## 3. The results

Table 2 (S1 Table) presents the estimation results, using as outcome the number of pregnancy-related deaths (the probability of pregnancies ending in death) associated with a one percentage point (p.p) increase in the percent of citizens who reported having paid for a bribe, gave a gift, or did a favour for police officers or governmental officials in last 12 months. We, first in column 1, adjust for country and year fixed-effects only. Subsequently, in column 2, we adjust for maternal and pregnancy-related characteristics, as well as for time-varying region covariates. The significant and negative coefficient of Bribery^2^ suggests us that the association between the percentage of individuals having paid a bribery, gave a gift and/or a favour to officials and maternal mortality appears to be hump-shaped.

**Table 2:** Additional pregnancy-related associated with a 1 p.p increase in first-hand experience in bribery.

	*Dependent variable*:		
	Prevalence of Maternal Mortality		
	Bivariate (1)	Fixed Effects (2)	Fixed Effect (Quintiles) (3)
Bribes	0.074	0.162***	
	(-0.002, 0.149)	(0.067, 0.256)	
Bribes Squared	-0.002**	-0.002**	
	(-0.003, -0.0004)	(-0.003, -0.001)	
Second Quintile (Ref. First Quintile)			0.379
			(-0.767, 1.525)
Third Quintile (Ref. First Quintile)			1.696**
			(0.463, 2.929)
Fourth Quintile (Ref. First Quintile)			1.853**
			(0.556, 3.149)
Fifth Quintile (Ref. First Quintile)			1.586*
			(0.204, 2.968)
Primary Education (Ref. No Education)		1.889***	1.924***
		(1.188, 2.590)	(1.222, 2.627)
Secondary Education or Higher (Ref. No Education)		-1.900**	-1.910**
		(-3.092, -0.709)	(-3.109, -0.710)
Antenatal Care or Skilled Delivery Assistance		-3.012***	-3.026***
		(-4.164, -1.860)	(-4.185, -1.867)
The Baby was born in a Facility (Ref. Born at Home)		-10.006***	-10.013***
		(-10.707, -9.304)	(-10.715, -9.310)
The Mother lives in a Rural Area (Ref. Urban Area)		0.274	0.274
		(-0.807, 1.354)	(-0.808, 1.356)
GFR		-0.004	-0.004
		(-0.018, 0.009)	(-0.017, 0.009)
log(GNI per capita)		-0.533	-0.487
		(-2.131, 1.064)	(-2.089, 1.114)
Observations	470,229	470,229	470,229
Controls	No	Yes	Yes
Country FE	No	Yes	Yes
Year FE	No	Yes	Yes

Authors’ elaboration on merged data for 17 SSA countries from the DHS and Afrobarometer for deliveries which took place over the period 2002–2018. We regress, through a linear probability model, the dummy variable equal to 1000 if the mother died (survived) during pregnancy or died within two months of childbirth on: the percentage of people reporting to have experience in bribery at the regional level (bribery) and the corresponding quadratic term; the logged regional Gross National Income per capita (GNI); the regional general fertility rate (GFR); the mother’s characteristics; pregnancy characteristics; year; and country fixed effect. Coefficients are multiplied by 1000. Educational attainment, urban-versus-rural residence, whether the baby was delivered at a facility, and antenatal care or skilled delivery assistance are imputed from the modal value for each sub-national-region-cohort, and are attached to every woman in the sample. Standard errors are robust to heteroscedasticity and clustered at the local level. 95% confidence intervals in brackets

* p<0.05,

** p<0.01,

*** p<0.001

**Fig 1:**
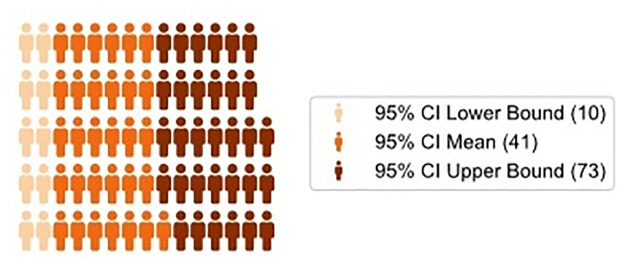
Association of first-hand experience in bribery with MM. Coefficients represent increase in the Number of Pregnancy-Related Deaths out of 1,000 Pregnancy-Related Deaths Associated with a 10 p.p. increase in First-Hand Experience in Bribery. Note: Authors’ elaboration on merged data for 17 SSA countries from the DHS and Afrobarometer for the 2002–2018 period. Each figure represents one fewer (blue) or additional (orange) pregnancy-related death every 1,000 pregnancy-related deaths if the prevalence of first-hand experience with bribery increased by 10 p.p. The reference level is the average level of maternal mortality among regions in the first quintile of the bribery distribution, corresponding to 8.93 ‰ in our sample. We regress through a linear probability model the dummy variable equal to 1,000 –if the mother died during the pregnancy or within two months of delivery–on the percentage of people reporting to have experience in bribery at the regional level (bribery) and the corresponding quadratic term, year and country fixed-effects, the mother’s characteristics, pregnancy characteristics, the regional Human Development Index (HDI) and the General Fertility Rate (GFR). Estimations are robust to heteroscedasticity and clustered at local level. We converted the estimation into a percentage variation in the number of pregnancy-related associated with a 10 p.p. increase in bribery.

To better illuminate the relevance of these estimates, Fig 1 displays the per-thousands estimated increase in the number of pregnancy-related deaths, compared the average in our sample corresponding to 8.6 ‰, increase in pregnancy-related deaths with a 10 p.p increase in the percentage of citizens’ reporting to have paid a bribe, gave a gift or a favour to an official in the last 12 month. It is worth noting that such an increase is experienced by 21.3% of our sample.

Each figure represents one fewer (blue) or additional (amber) pregnancy-related death. Darker (lighter) shades represent upper (lower) bounds. It is worth pointing out that we found only an increase in the associated mortality assessed to a level of 41 [95% CI: 10–73] additional deaths for every 1,000 pregnancy-related deaths compared to the average in our sample.

Turning to other covariates, presented in S1 Table, education erodes the detrimental effect of corruption against MM, as women living in areas with higher levels of educational attainment are less likely to die during pregnancy or within two months of birth [1, 45, 46]. Likewise, healthcare—proxied by the attendance of skilled health personnel during delivery/termination and antenatal care during pregnancy—significantly reduces MM [3, 4]. The place of delivery is not statistically significant, whereas the association between MM and HDI is negative. Our results also show that areas with higher levels of fertility are negatively associated with MM, though this is likely driven by the fact that the GFR ignores the different age structure of the population across different regions.

**Fig 2:**
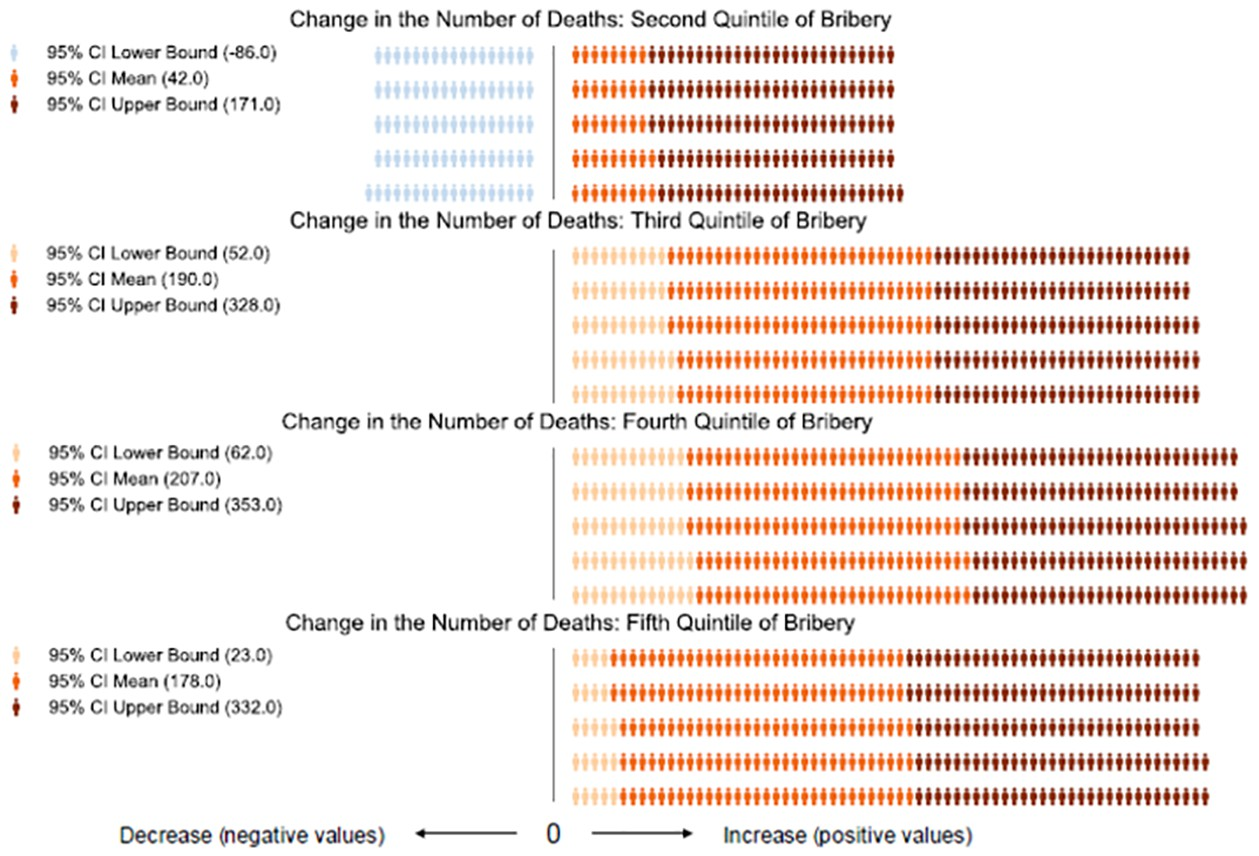
Association of first-hand experience in bribery with MM, estimation using bribes as a series of categorical variables rather than a continuous one. Coefficients represent Variation in Maternal Mortality, out of 1,000 Pregnancy-Related Deaths, compared to the Average Prevalence of Maternal Mortality in Regions within the First Quintile of the Bribery Distribution. Note: Authors’ elaboration on merged data for 17 SSA countries from the DHS and Afrobarometer for the 2002–2018 period. We regress through a linear probability model the dummy variable equal to 1000 –if the mother died during pregnancy or within two months of giving birth–on a series of dummies. These represent: the quantile distribution of bribery at the regional level; year and country fixed-effects; mother’s characteristics; pregnancy characteristics; the regional logged Gross National Income per capita (GNI); and the General Fertility Rate (GFR). Estimations are robust to heteroscedasticity and cluster at the local level. Each person represents one fewer (blue) or an additional (orange) pregnancy-related death for every 1,000 pregnancy-related deaths. The baseline is the average prevalence of life-ending pregnancies, corresponding to 8.6 ‰ in our sample. For example, the figure indicates that region-years falling in the third quintile of the bribery distribution have on average 190 additional deaths every 1000 when compared with regions within the first quintile.

To assess the robustness of our results we present a series of sensitivity tests. First, to ensure that our findings are not driven by the quadratic functional form, we replicate them by using, as the main explanatory variables, a series of dummies corresponding to the quintile—from the least corrupt to the most corrupt—of bribery-distribution for the region where the women gave birth. The results are presented in Table 2 column 3, where we give only the coefficient of each quintile, while the results are presented graphically in Fig 2. In S2 Table we present, instead, all the coefficients, using as reference category the least corrupted quintile. Again, each figure represents one fewer (blue) or additional (orange) pregnancy-related death out of every 1,000 pregnancy-related deaths. Darker (lighter) shades represent upper (lower) bounds. For example, Fig 2 indicates that, compared to regions in the first quintile of the bribery distribution, those in the third quintile have 190 [95% CI: 52, 328] additional deaths for every 1,000 pregnancies-related deaths. The results show that MM is positively associated with bribery. The association appears to be non-linear with the third and the fourth quintiles having the largest magnitude in the association. S1 Fig presents the relationship graphically.

### 3.1 The healthcare system as a moderator

**Fig 3:**
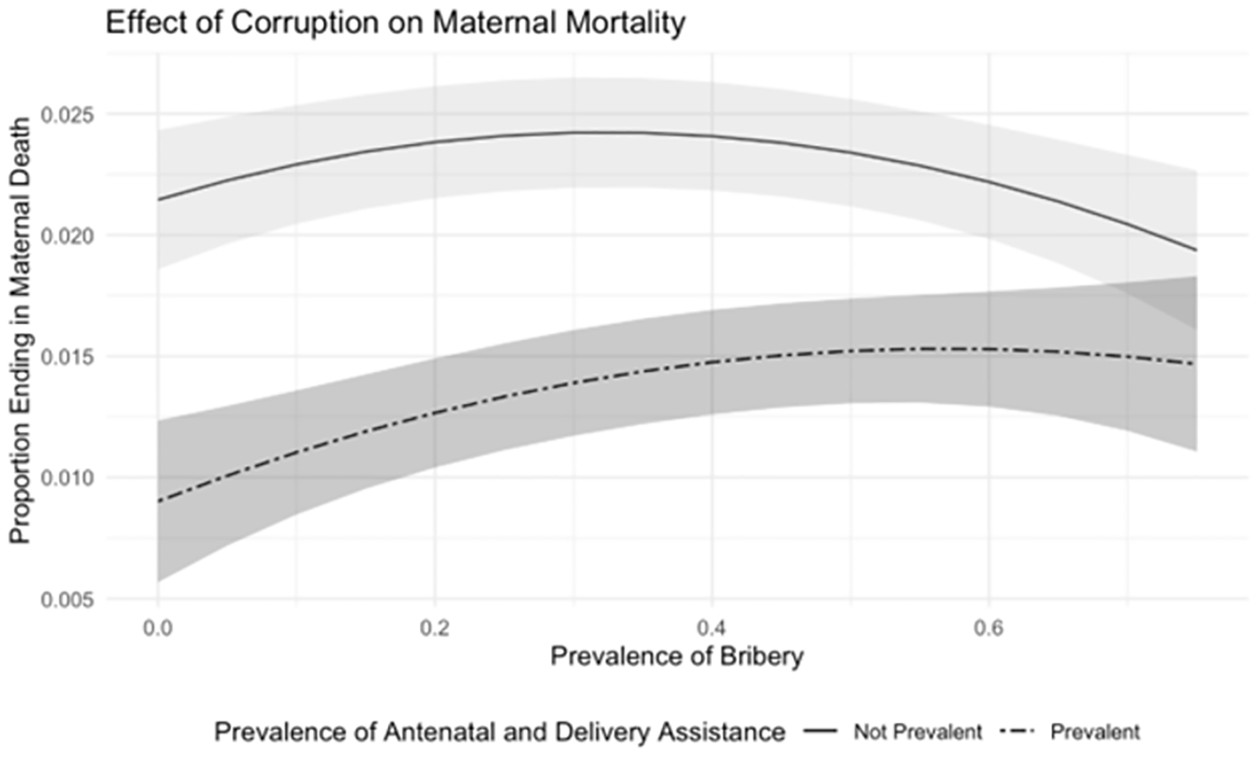
Association of first-hand experience in bribery with MM, the moderating role of the healthcare system. Notes: Authors’ elaboration on merged data for 17 SSA countries from the DHS and Afrobarometer for the 2002–2018 period. We regress through a linear probability model the dummy variable equal to 1,000 if the mother died during the pregnancy or within two months of giving birth on: the percentage of people reporting experiences in bribery at the regional level (bribery) and its quadratic term (bribery^2^); year and country fixed-effects; mother’s characteristics; pregnancy characteristics; the regional Human Development Index (HDI); the General Fertility Rate (GFR) and Bribery interaction; and whether antenatal care and/or being assisted during the delivery by skilled healthcare personnel was prevalent or not in the area where the mother lived in the delivery year. Standard errors are robust to heteroscedasticity and are clustered at local level.

It is of interest to understand if the association between the two variables under examination, namely MM and the percentage of citizens’ reporting to have paid a bribe/gave a gift or a favour to official in the last 12 months, changes according to the quality of the healthcare system. Our proxy of the quality of the healthcare system captures antenatal care or skilled birth attendants, specifically whether antenatal care and/or being assisted during the delivery by skilled healthcare personnel was prevalent or not in the area where the mother lived in the delivery year. The two proxies of the healthcare system quality are binary variables. We interacted the variable measuring bribery prevalence with healthcare system quality, thereby testing the moderator role of the healthcare system for this association. Fig 3 presents the results graphically by providing a set of predictions. The vertical axis represents the MM, whereas the horizontal axis represents the percentage of individuals with first-hand experience in bribery. All predictions include a 95% confidence interval. Predictions are then made for the two types of healthcare systems: one where neither antenatal care nor deliveries are attended by skilled healthcare personnel (solid line); the other were antenatal care or deliveries tend to benefit from skilled healthcare personnel (dashed line). The figure shows that, with low levels of bribery, the two systems differ starkly in terms of MM, with the system with prevalent antenatal care/ skilled birth attendants being better in MM terms. Whereas when bribery becomes more prevalent the gap between the two healthcare systems shrinks and the difference between the two systems becomes negligible. In other words, when the percentage of citizens’ who have paid a bribery/gave a gift or favor to official in the region is very low, MM is significantly lower (the confidence intervals do not overlap) in the regions where it is prevalent to observe either antenatal care and/or skilled birth personnel attending the delivery. Conversely, when the level of corruption is high, the estimates, where and where is not prevalent to observe either antenatal care and/or skilled birth personnel attending the delivery, cannot be distinguished as their estimate and their confidence intervals tend to overlap. We see, then, how corruption erodes the comparative advantage in terms of MM, and in terms of clinical interventions.

### 3.2 Using different proxies for corruption

**Fig 4:**
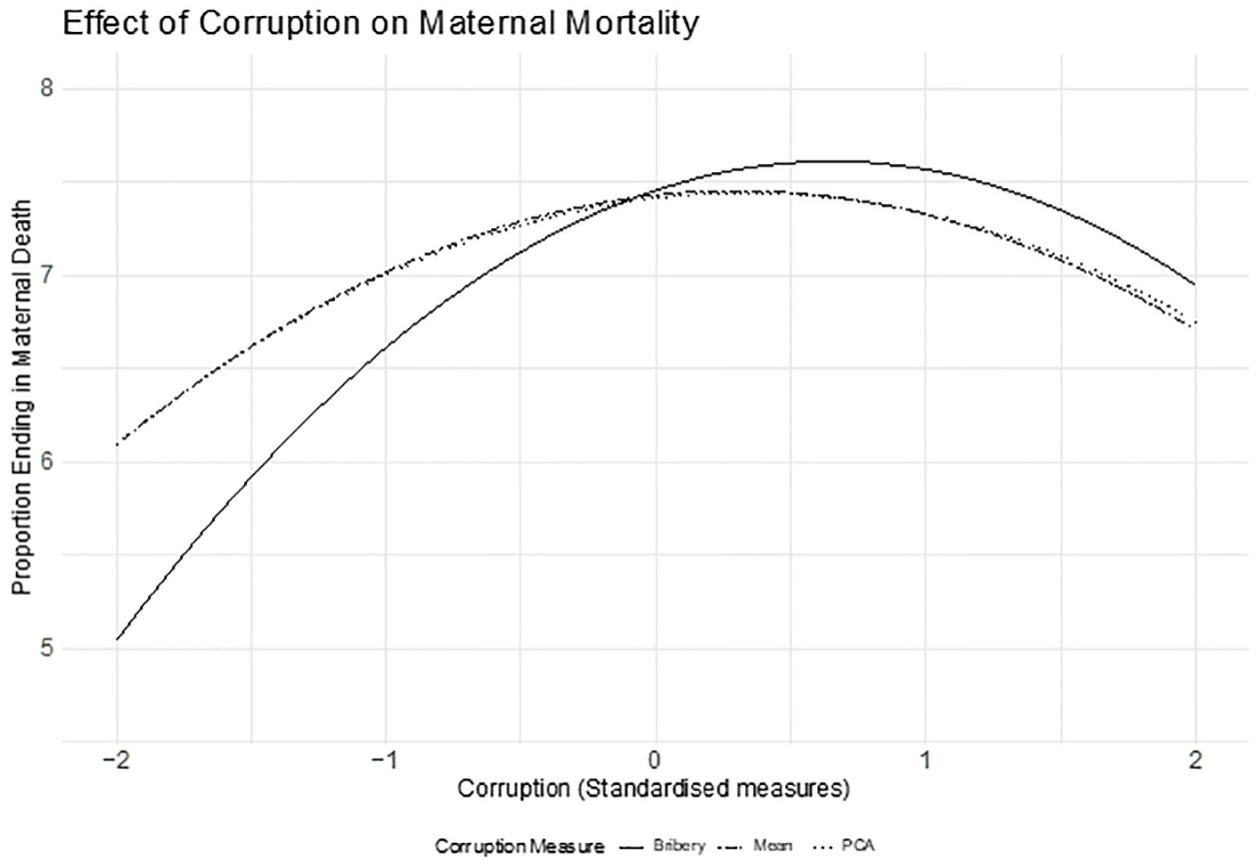
Association of first-hand experience in bribery with MM, using alternative methods of corruption. Notes: Authors’ elaboration on merged data for 17 SSA countries from the DHS and Afrobarometer for the 2002–2018 period. We regress through a linear probability model the dummy variable equal to 1000 if the mother died during the pregnancy or within two months of giving birth on: the percentage of people reporting experiences in bribery at the regional level (bribery) and its quadratic term (bribery^2^); year and country fixed-effects; mother’s characteristics; pregnancy characteristics; the regional Human Development Index (HDI); the General Fertility Rate (GFR) and Bribery interaction; and whether in the area where the mother lived on the year she gave birth antenatal care and/or being assisted during the delivery by skilled healthcare personnel was prevalent or not. Standard errors are robust to heteroscedasticity and are clustered at the local level. The confidence intervals reflect exclusively uncertainty around the coefficients on bribery.

Corruption is a highly complex concept not limited to bribery. As described in Section 2.1, we employ two alternative measures: the first is the principal component of four variables capturing perceptions of corruption, whereas the second is the simple average of the same four variables. To carry out a meaningful comparison, we standardized these two alternative measures and the original measure by subtracting their means and dividing them by their standard deviation. Fig 4 presents the comparison between the three measures graphically. The solid line represents bribery, the dashed line represents the alternative measure created, averaging out the four perceived corruption variables, and the dotted line depicts the alternative measure created through the principal component analysis. We present the relationship between maternal mortality and corruption using the three measures with 95% confidence intervals referring only to the uncertainty around the coefficients. The relationship appears rather similar and does not change the conclusions in this article. The only difference is that the alternative measures of corruption have larger confidence intervals, which is not unexpected. As we noted before, perceived corruption is a noisier measure compared to recordings of actual experienced episodes of bribery.

## 4. Discussion

As recognized by the SDGs, MM remains a major public health challenge especially in low- and middle-income countries. This is also a social justice issue, as its impact is spread unevenly across the socioeconomic spectrum, with the poorest facing the highest burden [47–49]. The causes for the persistence of this challenge are complex and difficult to determine and, only recently, have scholars and international organizations started to investigate them [34–36]. This study moves the debate forward by examining how corruption is coupled with MM. To this end, we used as a proxy for corruption the percentage of citizens’ who paid a bribe/gave a gift, or did a favour for governmental officials or the police in the last 12 months at regional level and we correlated that with the measure of MM. Using data for 135 regions from 17 SSA countries, this article finds evidence that higher levels of corruption are consistently associated with a higher probability of dying during pregnancy or shortly afterwards.

Our results are corroborated by previous literature that suggest five potential avenues in which corruption can affect MM. The first concerns public spending on healthcare [50, 51]. Mauro finds evidence that corrupt politicians tend to spend more public resources on those sectors on which it is easier to levy larger bribes [50]. However, given the intrinsic illegal nature of bribes, the ensuring need for secrecy imply that the politician will also choose sectors that are more difficult to monitor, such as high-technology goods, and the military sectors. Mauro also finds a negative effect on the healthcare sector as the opportunity to collect bribes on staff salaries is very limited [50]. Similar results for developing countries have been found by Delavallade [52]. Whereas Liang and Mirelman, using a dataset covering 120 countries–including both high-income and developing countries–come up with mixed results [51]. The authors find that the correlation between corruption and healthcare spending is positive in high income countries, while it is negative for low middle income ones [51]. Second, corruption reduces government revenues, which, in turn, leads to lower publicly provided services [53]. Third, corruption slows growth [54–56]. Corrupt governments tend to slow down governmental processes to increase opportunities for new bribes. Widespread corruption is, according to several cross-sectional studies, one of the leading reasons for the differential development of high-income countries *vis-à-vis* low or middle-income ones [54–56]. It appears to be associated with monopolies [57], and talent misallocation [58]. Moreover, very corrupt countries are less likely to attract innovation and entrepreneurship [58]. Fourth, corruption increases income inequalities [59], as low income groups will pay a much higher proportion on bribes compared to their better-off peers, which in turn might lead to tax evasion and hence to lower government revenues [53]. Fifth, corruption is negatively correlated with trust in public care [60], Radin, using data from Croatia, finds that citizens’ context and experience with corruption may transfer to their perception of the integrity of the healthcare system [60].

There are several ways to reduce MM. First and foremost, by improving health systems for timely access to skilled birth attendance, antenatal care, and emergency obstetric and neonatal care (when needed) for pregnant women [61]. A possibility to deliver such improvement, might be sought as in the case for Uganda by engaging with the private providers to expand the sector and/or further improve quality of the service, strengthen referral systems [62]. The second is through progress on another SDG: female empowerment or gender equality. This includes increasing women’s agencies and their ability to make strategic life choices, such as the use of modern contraception. Robust evidence shows that corruption matters in terms of an unmet need for modern contraception: corruption interferes with supply-chain quality and the diffusion of healthcare centers in rural areas, and it has a clear link to women’s awareness of access to and the availability of contraception [63]. Likewise, increasing the presence of women in politics [64]—across different decision-making arenas—may foster services which have been neglected but which affect MM. These include maternity care and access to safe abortions [65].

There are three important limitations to our work. First, our study is not experimental in design. Identification is based on a fixed-effect estimation strategy, though compared to previous studies, we do include country and sub-national regional fixed effects, which means that estimates derive from within sub-national variation. That said, we cannot claim causality as is done when implementing an experimental design. The study provides evidence on the correlation between bribery and MM at a fine-grained level, i.e. the pregnancy level, for a large set of countries over a fifteen-year period. Second, unfortunately only limited data are available for women who died during pregnancy. Therefore, some of the conclusions we draw might, as a result, be affected. However, the paucity of data has allowed us to focus on local systems rather than on the individual, providing new evidence on the role of sub-national systems on MM. This is of relevance for understanding MM and for providing local policymakers with appropriate tools. Third, our proxy of corruption does not focus on healthcare providers, which according to an attentive reader might be more relevant. Even though the Afrobarometer survey more recently included measures to understand how often individuals were forced to pay bribes for getting medicine or medical attention (wave 3) or get a treatment at a local health clinic or hospital (wave 5) or for treatment at public clinic or hospital (wave 6), those questions all have a slightly different wording and therefore they cannot be used to capture time and regional trends in bribery. Moreover, using them as main explanatory variable would lead to a large drop in our sample size. However, we believe that would be an interesting question for future research.

Our results suggest that investment in health interventions to curb MM, should be made in conjunction with measures to control and reduce corruption. Such multiple-front policy actions lie at the core of the SDG ‘system approach’—that is, developing parallel and concerted efforts in addressing interconnected policy problems. Progress on these complex problems will spur benefits in development and poverty reduction.

## Supporting information

S1 Fig Prevalence of life-ending pregnancies across 17 SSA countries. Note: Numbers represent number of life-ending pregnancies out 1,000 pregnancies.
(TIF)

S2 Fig **a**. First-hand Experience in Bribery across 17 SSA Countries–The unit of observation is the pregnancy, so women having more than one baby during the period 2002–2018 are counted more than once. **b**. First-hand Experience in Bribery across 17 SSA Countries—The unit of observation is the sub-national region.
(ZIP)

S3 Fig Geographical distribution of first-hand experience in bribery over time. Note: Authors’ elaboration on merged data for 135 regions covering 17 SSA countries from Afrobarometer for 2002–2016 period. The quintiles are computed separately for each Afrobarometer round to highlight changes in the relative position of different regions. The map is computed through open sources data, specifically “GDL Shapefile V6” from the Global Data Lab: https://globaldatalab.org/mygdl/downloads/shapefiles/.
(TIF)

S1 Table Variation in the probability of a life-ending pregnancy associated with first-hand experience in bribery. Notes: Authors’ elaboration on merged data for 17 SSA countries from the DHS and Afrobarometer for deliveries which took place over the period 2002–2018. We regress, through a linear probability model, the dummy variable equal to 1(0) if the mother died (survived) during pregnancy or died within two months of childbirth on the percentage of people reporting to have experience in bribery at the regional level (first column) or a set of dummy variables for the quintiles of the bribery distribution (second column). Controls are: the logged regional Gross National Income per capita (GNI), regional general fertility rate (GFR), mother’s characteristics, pregnancy characteristics, year and country fixed effect. Educational attainment, urban-versus-rural residence, whether the baby was delivered at a facility, and antenatal care or skilled delivery assistance are imputed from the modal value for each sub-national-region-cohort, and attached to every woman in the sample. Standard errors are robust to heteroscedasticity and clustered at local level. 95% confidence intervals in brackets + p<0.1, * p<0.05, ** p<0.01, *** p<0.001.
(DOCX)

S2 Table Variation in the probability of a life-ending pregnancy associated with first-hand experience in bribery (multi level models). (DOCX)

S3 Table Descriptive statistics. Data availability and number of observations for both maternal mortality and bribery at country level.
(DOCX)
