# Correction: Does bribery increase maternal mortality? Evidence from 135 Sub-Saharan African regions

**DOI:** 10.1371/journal.pgph.0003570

**Published:** 2024-07-24

**Authors:** Veronica Toffolutti, Eugenio Paglino, Alexandros Kentikelenis, Letizia Mencarini, Arnstein Aassve

In the Introduction section, there is an error in the last paragraph. The correct sentence is: The paper proceeds as follows we present the methodological approach in section 2, the results in section 3, and finally we offer our conclusions.

The heading of section 2, subsection 2.1 and subdivision 2.1.1 appears incorrectly in the article. The correct heading is:

2. The methodological approach

2.1. Data sources
