# Supplementary figures and images for: Does bribery increase maternal mortality? Evidence from 135 Sub-Saharan African regions
Source: PLOS Glob Public Health. 2023 Dec 4;3(12):e0000847. doi: 10.1371/journal.pgph.0000847 (PMC10695367; doi:10.1371/journal.pgph.0000847)

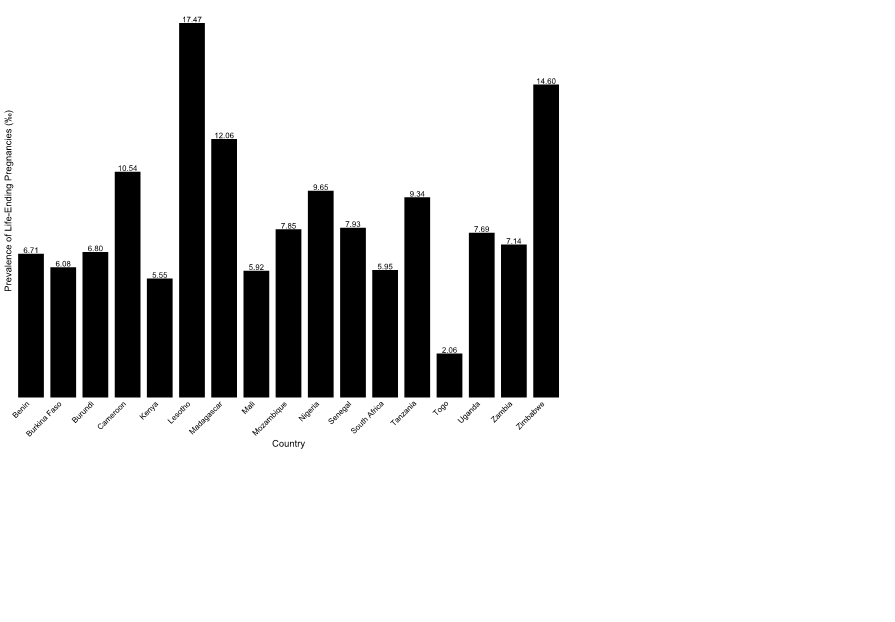

Supplement: S1 Fig — Note: Numbers represent number of life-ending pregnancies out 1,000 pregnancies. (TIF) [file pgph.0000847.s001.tif]

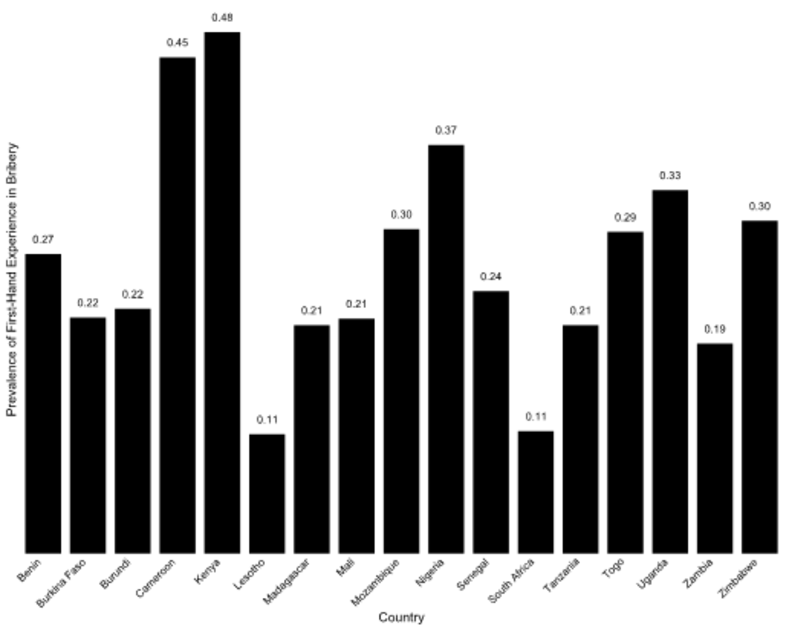

Supplement: S2 Fig — a. First-hand Experience in Bribery across 17 SSA Countries–The unit of observation is the pregnancy, so women having more than one baby during the period 2002–2018 are counted more than once. b. First-hand Experience in Bribery across 17 SSA Countries—The unit of observation is the sub-national region. (ZIP) [file pgph.0000847.s002.zip › S2b_Fig.tif]

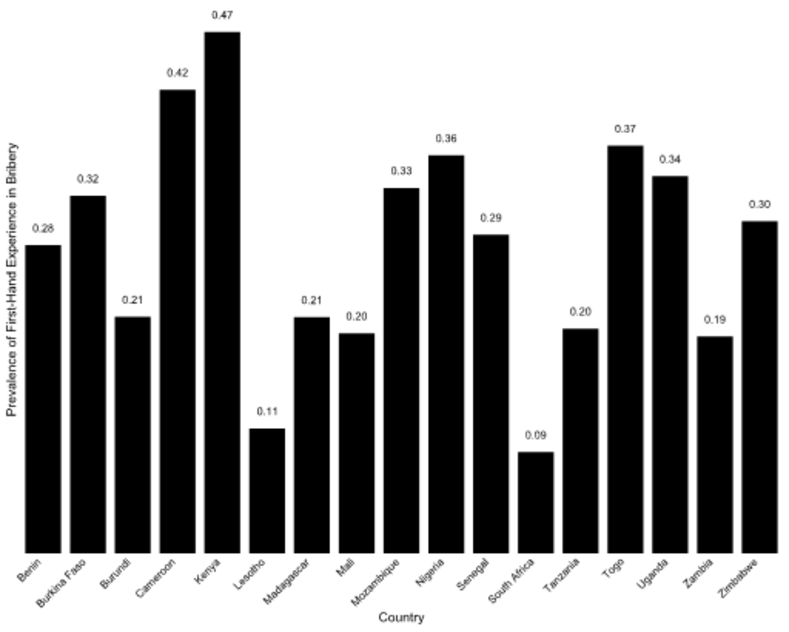

Supplement: S2 Fig — a. First-hand Experience in Bribery across 17 SSA Countries–The unit of observation is the pregnancy, so women having more than one baby during the period 2002–2018 are counted more than once. b. First-hand Experience in Bribery across 17 SSA Countries—The unit of observation is the sub-national region. (ZIP) [file pgph.0000847.s002.zip › S2a_Fig.tif]

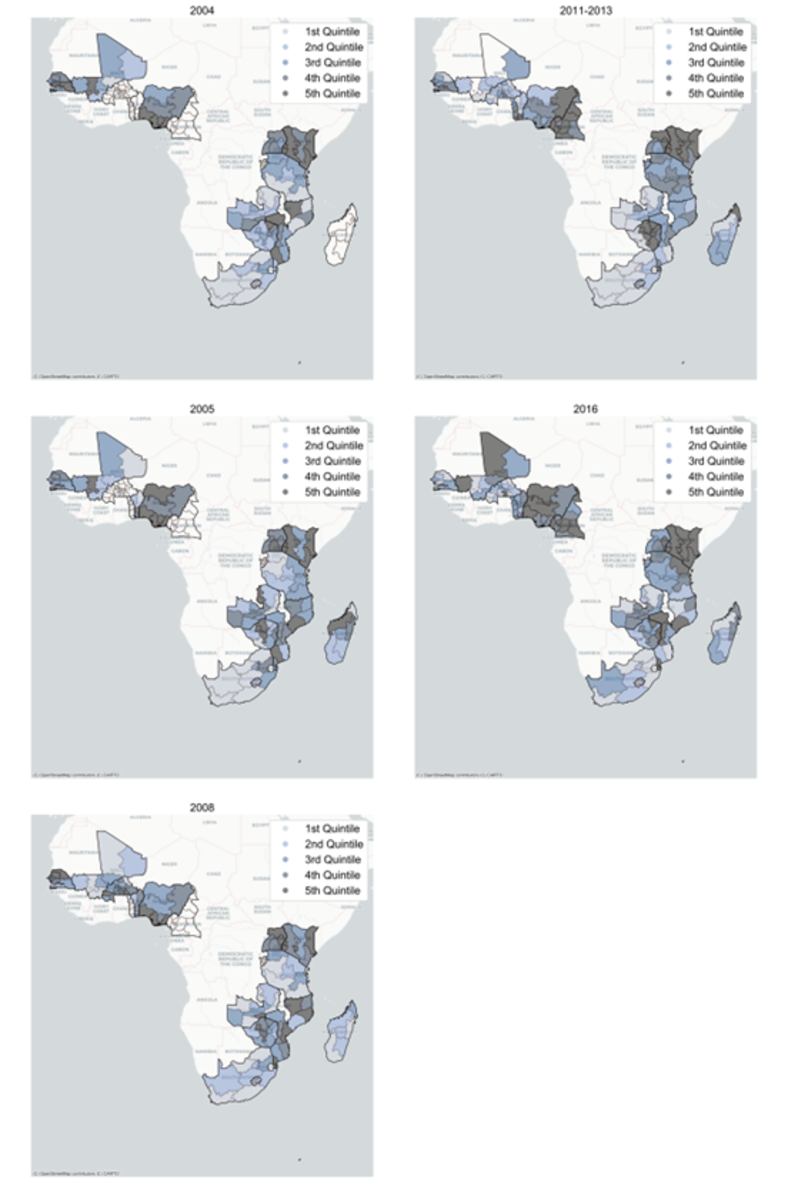

Supplement: S3 Fig — Note: Authors’ elaboration on merged data for 135 regions covering 17 SSA countries from Afrobarometer for 2002–2016 period. The quintiles are computed separately for each Afrobarometer round to highlight changes in the relative position of different regions. The map is computed through open sources data, specifically “GDL Shapefile V6” from the Global Data Lab: https://globaldatalab.org/mygdl/downloads/shapefiles/. (TIF) [file pgph.0000847.s003.tif]
