# Supplementary material for: Does bribery increase maternal mortality? Evidence from 135 Sub-Saharan African regions
Source: PLOS Glob Public Health. 2023 Dec 4;3(12):e0000847. doi: 10.1371/journal.pgph.0000847 (PMC10695367; doi:10.1371/journal.pgph.0000847)
Supplement: S1 Table — Notes: Authors’ elaboration on merged data for 17 SSA countries from the DHS and Afrobarometer for deliveries which took place over the period 2002–2018. We regress, through a linear probability model, the dummy variable equal to 1(0) if the mother died (survived) during pregnancy or died within two months of childbirth on the percentage of people reporting to have experience in bribery at the regional level (first column) or a set of dummy variables for the quintiles of the bribery distribution (second column). Controls are: the logged regional Gross National Income per capita (GNI), regional general fertility rate (GFR), mother’s characteristics, pregnancy characteristics, year and country fixed effect. Educational attainment, urban-versus-rural residence, whether the baby was delivered at a facility, and antenatal care or skilled delivery assistance are imputed from the modal value for each sub-national-region-cohort, and attached to every woman in the sample. Standard errors are robust to heteroscedasticity and clustered at local level. 95% confidence intervals in brackets + p<0.1, * p<0.05, ** p<0.01, *** p<0.001. (DOCX) [file pgph.0000847.s004.docx]

|  | Bivariate | Fixed Effects | Fixed Effect (Quintiles) |
| --- | --- | --- | --- |
| Bribes | 0.0735 | 0.1616 * |  |
|  | [-0.0022; 0.1492] | [ 0.0670; 0.2562] |  |
| Bribes^2 | -0.0015 * | -0.0020 * |  |
|  | [-0.0026; -0.0004] | [ -0.0033; -0.0008] |  |
| Quantiles of Bribery (Reference: First Quintile) |  |  |  |
| Second Quintile |  |  | 0.3787 |
|  |  |  | [ -0.7673; 1.5247] |
| Third Quintile |  |  | 1.6961 * |
|  |  |  | [ 0.4628; 2.9294] |
| Fourth Quintile |  |  | 1.8526 * |
|  |  |  | [ 0.5560; 3.1492] |
| Fifth Quintile |  |  | 1.5865 * |
|  |  |  | [ 0.2044; 2.9685] |
| Modal Education Level (Reference: No Education) |  |  |  |
| Primary Education |  | -1.9003 * | -1.9095 * |
|  |  | [ -3.0917; -0.7089] | [ -3.1087; -0.7103] |
| Secondary Education or Higher |  | -3.0123 * | -3.0260 * |
|  |  | [ -4.1643; -1.8602] | [ -4.1852; -1.8667] |
| Antenatal Care or Skilled Delivery Assistance |  | -10.0055 * | -10.0127 * |
|  |  | [-10.7074; -9.3037] | [-10.7153; -9.3101] |
| The Baby was born in a Facility (Reference: The Baby was Born at Home) |  | 1.8891 * | 1.9243 * |
|  |  | [ 1.1878; 2.5903] | [ 1.2216; 2.6269] |
| The Mother lives in a Rural Area (Reference: Woman Lives in a Urban Area) |  | 0.2739 | 0.2739 |
|  |  | [ -0.8065; 1.3543] | [ -0.8079; 1.3557] |
| GFR |  | -0.0044 | -0.0039 |
|  |  | [ -0.0177; 0.0089] | [ -0.0172; 0.0095] |
| log(GNI per capita) |  | -0.5334 | -0.4874 |
|  |  | [ -2.1309; 1.0640] | [ -2.0892; 1.1144] |
| Controls | No | Yes | Yes |
| Year FE | No | Yes | Yes |
| Country FE | No | Yes | Yes |
| Num. obs. | 470229 | 470229 | 470229 |
