# Supplementary material for: Does bribery increase maternal mortality? Evidence from 135 Sub-Saharan African regions
Source: PLOS Glob Public Health. 2023 Dec 4;3(12):e0000847. doi: 10.1371/journal.pgph.0000847 (PMC10695367; doi:10.1371/journal.pgph.0000847)
Supplement: S2 Table — (DOCX) [file pgph.0000847.s005.docx]

|  | Linear | Quadratic | Linear (slope) | Quadratic (slope) |
| --- | --- | --- | --- | --- |
| Time | -0.3588 * | -0.8211 | -0.3040 * | -0.8677 |
|  | [-0.6288; -0.0887] | [-1.6946; 0.0524] | [-0.5742; -0.0338] | [-1.8220; 0.0866] |
| Time^2 |  | 0.0273 |  | 0.0525 |
|  |  | [-0.0298; 0.0844] |  | [-0.0240; 0.1291] |
| Bribes | 0.0924 | 0.0559 |  |  |
|  | [-0.0513; 0.2361] | [-0.0944; 0.2062] |  |  |
| Bribes^2 | -0.0028 * | -0.0027 * |  |  |
|  | [-0.0048; -0.0007] | [-0.0048; -0.0005] |  |  |
| Time * Bribes | 0.0145 * |  | 0.0192 * | 0.0630 * |
|  | [ 0.0069; 0.0222] |  | [ 0.0063; 0.0321] | [ 0.0138; 0.1122] |
| Time * Bribes^2 |  |  | -0.0002 * | -0.0011 * |
|  |  |  | [-0.0004; -0.0001] | [-0.0017; -0.0004] |
| Time^2 * Bribes |  | -0.0007 |  | -0.0044 |
|  |  | [-0.0025; 0.0011] |  | [-0.0087; 0.0000] |
| Time^2 * Bribes^2 |  |  |  | 0.0001 * |
|  |  |  |  | [ 0.0000; 0.0001] |
| AIC | 5587685.7430 | 5587696.2942 | 5587672.7383 | 5587668.6707 |
| BIC | 5588061.8162 | 5588205.0990 | 5587949.2626 | 5587978.3780 |
| Log Likelihood | -2793808.8715 | -2793802.1471 | -2793811.3691 | -2793806.3354 |
| Controls | Yes | Yes | Yes | Yes |
| Num. obs. | 470229 | 470229 | 470229 | 470229 |
| Num. groups: GDLCode | 135 | 135 | 135 | 135 |
| Num. groups: country | 17 | 17 | 17 | 17 |
