# Supplementary material for: Does bribery increase maternal mortality? Evidence from 135 Sub-Saharan African regions
Source: PLOS Glob Public Health. 2023 Dec 4;3(12):e0000847. doi: 10.1371/journal.pgph.0000847 (PMC10695367; doi:10.1371/journal.pgph.0000847)
Supplement: S3 Table — Data availability and number of observations for both maternal mortality and bribery at country level. (DOCX) [file pgph.0000847.s006.docx]

|  | **2002** | **2003** | **2004** | **2005** | **2006** | **2007** | **2008** | **2009** | **2010** | **2011** | **2012** | **2013** | **2014** | **2015** | **2016** | **2017** | **2018** |
| --- | --- | --- | --- | --- | --- | --- | --- | --- | --- | --- | --- | --- | --- | --- | --- | --- | --- |
| **Benin** | N/A | N/A | N/A | N/A | N/A | N/A | 2008 | 2200 | N/A | 2311 | N/A | N/A | 2632 | 2474 | 2607 | 2903 | N/A |
| **Burkina Faso** | N/A | N/A | N/A | N/A | N/A | N/A | 2411 | 2416 | 2077 | N/A | N/A | N/A | N/A | N/A | N/A | N/A | N/A |
| **Burundi** | N/A | N/A | N/A | N/A | N/A | N/A | N/A | N/A | N/A | N/A | 2711 | 2497 | 2485 | 2703 | 2701 | N/A | N/A |
| **Cameroon** | N/A | N/A | N/A | N/A | N/A | N/A | N/A | N/A | N/A | N/A | N/A | N/A | 2002 | 1936 | 1954 | 1971 | 1626 |
| **Kenya** | N/A | 5395 | 5349 | 5082 | 5598 | N/A | 6293 | N/A | N/A | 4394 | 4088 | N/A | 2687 | N/A | N/A | N/A | N/A |
| **Lesotho** | N/A | 1472 | 1530 | 1132 | 1144 | N/A | 1527 | 1429 | 606 | N/A | 589 | 666 | 494 | N/A | N/A | N/A | N/A |
| **Madagascar** | N/A | N/A | N/A | 2394 | 2588 | N/A | 2561 | N/A | N/A | N/A | N/A | N/A | N/A | N/A | N/A | N/A | N/A |
| **Mali** | 5071 | 4704 | N/A | 4509 | 4276 | N/A | 2369 | 2354 | 2348 | N/A | 2087 | N/A | 1218 | 1209 | 1162 | 1256 | 1048 |
| **Mozambique** | N/A | N/A | N/A | N/A | N/A | N/A | 2178 | 2140 | 2563 | N/A | N/A | N/A | N/A | N/A | N/A | N/A | N/A |
| **Nigeria** | N/A | 15115 | 15306 | 16771 | 17060 | N/A | 17683 | 11162 | 13170 | N/A | 13957 | 9228 | 6915 | 6934 | 6493 | 6491 | 5971 |
| **Senegal** | 4211 | 4473 | N/A | 3086 | 2200 | N/A | 2375 | 2468 | 2632 | N/A | N/A | N/A | N/A | N/A | N/A | N/A | N/A |
| **South Africa** | N/A | N/A | N/A | N/A | N/A | N/A | N/A | N/A | N/A | N/A | 739 | 714 | N/A | 706 | 530 | N/A | N/A |
| **Tanzania** | N/A | N/A | N/A | N/A | 330 | N/A | 3436 | 3644 | 2254 | N/A | 1946 | 2024 | 2263 | 2009 | N/A | N/A | N/A |
| **Togo** | N/A | N/A | N/A | N/A | N/A | N/A | N/A | N/A | N/A | N/A | 1426 | 1492 | N/A | N/A | N/A | N/A | N/A |
| **Uganda** | 4865 | 5206 | N/A | 5493 | 5210 | N/A | 4586 | 4328 | 4654 | N/A | 3058 | 3039 | N/A | 3110 | 2466 | N/A | N/A |
| **Zambia** | N/A | 4796 | 5029 | 5082 | 5637 | 5192 | N/A | 4619 | 4751 | 4463 | N/A | 4369 | 1564 | 1391 | 1484 | 1478 | 1269 |
| **Zimbabwe** | N/A | N/A | 2612 | 2826 | 1872 | 1967 | N/A | 2159 | 2645 | N/A | 1257 | 1201 | 1225 | 1004 | N/A | N/A | N/A |
